# PReS-FINAL-2200: Phenotype of V198M and Q703K NLRP3 variants

**DOI:** 10.1186/1546-0096-11-S2-P190

**Published:** 2013-12-05

**Authors:** V Messia, M Pardeo, R Nicolai, C Bracaglia, F De Benedetti, A Insalaco

**Affiliations:** 1 Pediatric Medicine-Rheumatology, Ospedale Pediatrico Bambino Gesù, Rome, Italy

## Introduction

The term CAPS (Cryopyrin-Associated Periodic Syndromes) identifies a spectrum of autoinflammatory diseases caused by heterozygous mutations of the CIAS1/NLRP3. Affected individuals may present three different phenotypes: Familial Cold Autoinflammatory Syndrome (FCAS), Muckle-Wells Syndrome (MWS) and CINCA syndrome, the most severe form of the clinical spectrum. Clinical manifestations include urticaria-like rash, recurrent fever, arthralgia, conjunctivitis; chronic aseptic meningitis, cerebral atrophy and bone malformations in the severe cases.

The term CAPS (Cryopyrin-Associated Periodic Syndromes) identifies a spectrum of autoinflammatory diseases caused by heterozygous mutations of the CIAS1/NLRP3. Affected individuals may present three different phenotypes: Familial Cold Autoinflammatory Syndrome (FCAS), Muckle-Wells Syndrome (MWS) and CINCA syndrome, the most severe form of the clinical spectrum. Clinical manifestations include urticaria-like rash, recurrent fever, arthralgia, conjunctivitis; chronic aseptic meningitis, cerebral atrophy and bone malformations in the severe cases.

## Objectives

To describe the long-term clinical course of a cohort of patients carrying two different low-penetrance NLRP3 mutations (V198M and Q703K).

## Methods

Six patients were identified carrying the NLRP3 V198M mutation (mean age 10,35 ± 4,73 years, 4 males and 2 females), and 5 patients were identified carrying the NLRP3 Q703K (mean age 9,72 ± 4,55 years, 3 males and 2 females). All were Caucasians.

## Results

In the V198M cohort the mean age at disease onset was 5,85 ± 4,08 years. All patients had symptoms consistent with recurrent inflammatory syndrome: 6/6 presented recurrent episodes of skin lesions and arthralgia, 4/6 of fever attacks, 3/6 of arthritis, 2/6 of headache and subcutaneous edema. One patient showed fatigue, conjunctivitis and recurrent abdominal pain. Half of the patients had a positive family history for recurrent inflammatory episodes. In 3 out of 6 patients the severity of phenotype and the persistence of elevated acute phase reactants, led to initiation of anti IL-1 therapy with immediate benefit. In the cohort of patients with Q703K variant the mean age at disease onset was 3,73 ± 3,33 years. All patients had skin rash, 4/5 patients presented recurrent fever, 3/5 arthralgia and myalgia, 2/5 subcutaneous edema, pharyngitis and lymphadenitis; 1 out of 5 patients had mild arthritis, headache and abdominal pain. Only in 1 case, symptoms were triggered or worsened by cold exposure. None of our patients had a family history relevant for autoinflammatory symptoms. Laboratory test showed no increase in acute phase reactants, with one exception. This patient presented also with recurrent fevers, treatment resistant epilepsy and carries an heterozygous MEFV mutation. She failed colchicine and anti IL-1 therapy was started with benefit.

## Conclusion

The pathogenic significance of these NLRP3 mutations is still discussed. In our experience patients carrying Q703K mutation appear to have a milder and self-limited phenotype than those with V198M variant in which therapy with IL-1 inhibitor drugs is often necessary. The factors that affect the pathogenic consequences of these variants are still to be established.

## Disclosure of interest

None declared.

